# Balancing public health priorities and inclusivity in medical education: a clarification on curriculum revisions

**DOI:** 10.1016/j.lansea.2025.100563

**Published:** 2025-03-22

**Authors:** Mirza Jahanzeb Beg, B. Sai Chaitanya Reddy, Manik Inder Singh Sethi

**Affiliations:** a Department of Psychology, Kumaraguru College of Liberal Arts and Science, Coimbatore, Tamil Nadu, India; b Telemedicine Centre, Nimhans Digital Academy, Department of Psychiatry, National Institute of Mental Health & Neurosciences, Bengaluru, India

The recent article by Singh et al. in *The Lancet* on India's revised Competency-Based Medical Education (CBME) curriculum raises important questions about inclusivity in medical education.^1^ While their concern for marginalized communities is valid, their approach exemplifies how academic discourse, when disconnected from practical implications, can inadvertently promote policy changes that compromise healthcare delivery and legal protections for vulnerable populations.

The authors’ conflation of disability rights and LGBTQIA+ healthcare competencies undermines their critique. These domains require distinct consideration in medical education, each with unique challenges, terminologies, and professional requirements. Furthermore, their criticism of medico-legal terminology, such as “sodomy,” reflects a misunderstanding of the intersection between medical education and legal frameworks. For instance, in cases of sexual violence, medical professionals must use precise, legally recognized terminology to ensure justice while maintaining sensitivity toward survivors. The current need is to address this by contextualizing terms like “sodomy” within a medico-legal framework, distinguishing consensual acts from criminal offenses—a balance essential for both legal clarity and compassionate care.

Recent legislative developments highlight the risks of rushed policy changes. The absence of provisions equivalent to Section 377 IPC in the Bharatiya Nyaya Sanhita (BNS), despite the Supreme Court's stance on non-consensual acts, demonstrates how pressure from academic discourse can create dangerous gaps in legal protections.^2^ Medical professionals, as both healthcare providers and expert witnesses, must retain the ability to document cases using terminology that ensures justice while delivering trauma-informed care.

The National Medical Commission's (NMC) approach to curriculum development reflects thoughtful evolution through stakeholder engagement. Consultations with LGBTQIA+ advocacy groups, disability rights organizations, and legal experts have shaped competencies that balance professional requirements with social progress. This collaborative process ensures that curriculum changes reflect both the evolving needs of medical education and the lived realities of marginalized communities.^3^

Medical education plays a pivotal role in addressing societal stigma. For example, a transgender individual's healthcare experience depends on their provider's clinical and cultural competency. The curriculum equips future doctors to navigate complex legal and social landscapes, ensuring equitable care while advocating for systemic change.

The Supreme Court's recent handbook on combating gender stereotypes exemplifies the measured approach needed in medical education. Immediate removal of essential medico-legal training risks undermining both healthcare delivery and the justice system. Instead, continuous evaluation and adaptation of the curriculum will ensure reforms align with social progress while maintaining professional standards.^4^

In conclusion, effective medical education reform must balance professional competency, legal necessity, social progress, and community needs. By advocating for evidence-based, incremental changes, we can equip future medical professionals to serve all communities effectively while driving systemic improvements in healthcare access and delivery.

## Contributors

All authors contributed equally to this manuscript. Specifically, Mirza Jahanzeb Beg, Dr. Manik Inder Singh Sethi, and B. Sai Chaitanya Reddy were involved in the literature search and writing of the manuscript. Each author has accessed and verified the underlying data reported in the manuscript.

## Declaration of interests

None.
